# Genome wide association study and genomic prediction for stover quality traits in tropical maize (*Zea mays* L.)

**DOI:** 10.1038/s41598-020-80118-2

**Published:** 2021-01-12

**Authors:** M. T. Vinayan, K. Seetharam, Raman Babu, P. H. Zaidi, M. Blummel, Sudha K. Nair

**Affiliations:** 1 Centro Internacional de Mejoramiento de Maíz y Trigo (CIMMYT), c/o ICRISAT, Patancheru, 502324 India; 2 International Livestock Research (ILRI), c/o ICRISAT, Patancheru, 502324 India; 3 Present Address: Corteva Agrisciences, Multi Crop Research Centre, Hyderabad, India

**Keywords:** Plant breeding, Plant sciences

## Abstract

Maize is rapidly replacing traditionally cultivated dual purpose crops of South Asia, primarily due to the better economic remuneration. This has created an impetus for improving maize for both grain productivity and stover traits. Molecular techniques can largely assist breeders in determining approaches for effectively integrating stover trait improvement in their existing breeding pipeline. In the current study we identified a suite of potential genomic regions associated to the two major stover quality traits—in-vitro organic matter digestibility (IVOMD) and metabolizable energy (ME) through genome wide association study. However, considering the fact that the loci identified for these complex traits all had smaller effects and accounted only a small portion of phenotypic variation, the effectiveness of following a genomic selection approach for these traits was evaluated. The testing set consists of breeding lines recently developed within the program and the training set consists of a panel of lines from the working germplasm comprising the founder lines of the newly developed breeding lines and also an unrelated diversity set. The prediction accuracy as determined by the Pearson’s correlation coefficient between observed and predicted values of these breeding lines were high even at lower marker density (200 random SNPs), when the training and testing set were related. However, the accuracies were dismal, when there was no relationship between the training and the testing set.

## Introduction

Recent studies have highlighted the potential of maize stover as an important source of fodder in livestock farming systems globally^1–4^. Crop residues provide major feed resources for livestock in low and middle income countries (LMCs). In India, maize is now the third most important crop after rice and wheat, and it is cultivated on over 8.7 million ha with 24.0 million ton of grain produced at an average yield of 2.6 tha^−1^^5^. Most of the increase has been recorded in non-traditional maize growing areas where it is grown as an irrigated crop for commercial purpose during the dry season. In these areas, maize is largely replacing sorghum, an important dual-purpose crop. The stover of sorghum is highly valued by livestock keepers and fodder traders^6,7^. Given the prevalent fodder shortage in India, maize stover would need to substitute for the loss in sorghum stover^8^. Increases in maize area have also been reported in East Africa, where there is a similar need to look into the fodder value of maize stover^2,9,10^. However, breeding programs focused on maize improvement primarily in regions of South Asia are directed largely towards grain improvement with diminutive focus on stover traits.

Improving the quality of maize stover has been addressed through conventional breeding strategies^8,11^. However, simultaneous improvement of stover and grain in maize would require repeat phenotyping for both grain yield and stover quality traits substantially increasing the conventional breeding costs and time per cycle. With the advent of molecular markers, maize breeding programs across several regions had made a paradigm shift and incorporated marker based technologies for various trait improvements in the breeding pipeline particularly due to its advantages with high throughput efficient selections and cost reduction of the breeding processes. With the advancements in molecular marker systems, the identification of candidate genomic regions that have significant effects on fodder quality, and deployment of these regions through breeding has become more feasible. Earliest reports for Quantitative trait loci (QTL) mapping for stover quality traits dates to 1997^12,13^. Several studies have since identified various QTLs for different silage traits in maize^14–23^. In an attempt to resynthesize the information on stover quality Truntzler et al.^23^ conducted a meta analysis on the combined results of 14 such studies in maize, and identified regions (QTLs) with significant importance and smaller confidence interval on chromosomes 1, 2, 3, 4, 5 and 9 for digestibility traits and cell wall component traits. In addition, studies with candidate genes had also identified specific regions associated with digestibility and/or cell wall synthesis on chromosomes 9, 4, 3 and 1 using a limited set of maize^24^ lines. However, QTL mapping approaches are often restricted by the limited recombination in the mapping population, that are widely different from the once observed in natural population^25^ and hence often have narrow applicability. Genome-wide association study (GWAS) is another powerful molecular tool for crop improvement, which has been successful in identifying genomic regions for plant height, root traits, flowering time and a range of disease resistance traits^26–28^. GWAS studies on stalk strength and stalk components have also been reported in maize^29–31^. GWAS on maize stover digestibility traits (IVOMD, NDF and ADF) have also been studied previously^32^ on a panel of 296 maize test crosses, that had revealed several significant genomic regions particularly on chromosome 3 (bin 3.05) and chromosome 9 (bin 9.08). However, validation of these genomic regions in an independent population or panel, and also with in the working germplasm of the breeding program is an important pre-requisite for their integration in marker assisted breeding. In addition, the loci identified for these complex traits often explained only a small portion of the observed phenotypic variation, which often restricts their applicability in breeding program^33^.

Reduction in the cost of genotyping and use of high-throughput genotyping facilities, has paved the way for deploying genomic selection strategies in routine breeding programs. Genomic selection has been widely practiced in animal breeding^34^ and is being utilized successfully in plant breeding as well^35–37^. However, successful integration of genomic selection with the breeding pipeline circumventing the need for conventional selection is determined largely based on the cost and time involved in phenotyping the trait as well as the genotypic prediction accuracy between the testing set and training set^38^. Prediction accuracy as determined by the correlation between the breeding value and the genomic estimated values of individuals, varies with different parameters^39^. Trait complexity and the degree of similarity between the training and testing sets are two such factors that determine the accuracy of prediction. Zhang et al.^40^ reported moderate to high accuracies when training and test populations were related. Morgante et al.^33^ further suggested that the accuracies can often go low in cases where the actual loci affecting the traits are far fewer than the molecular markers used and if large epistatic interactions are involved in a trait. Prediction accuracies are affected by the genetic architecture of the trait, marker densities, minor allelic frequencies and population relationships^41^. In this context the current study focused on (i) identifying genomic regions for two major stover quality traits through GWAS in an independent association panel and (ii) determining if a genomic prediction approach could be followed to identify non-phenotyped lines with good stover quality traits.

## Results

### Variability for stover quality traits in association mapping panel

**Figure 1:**
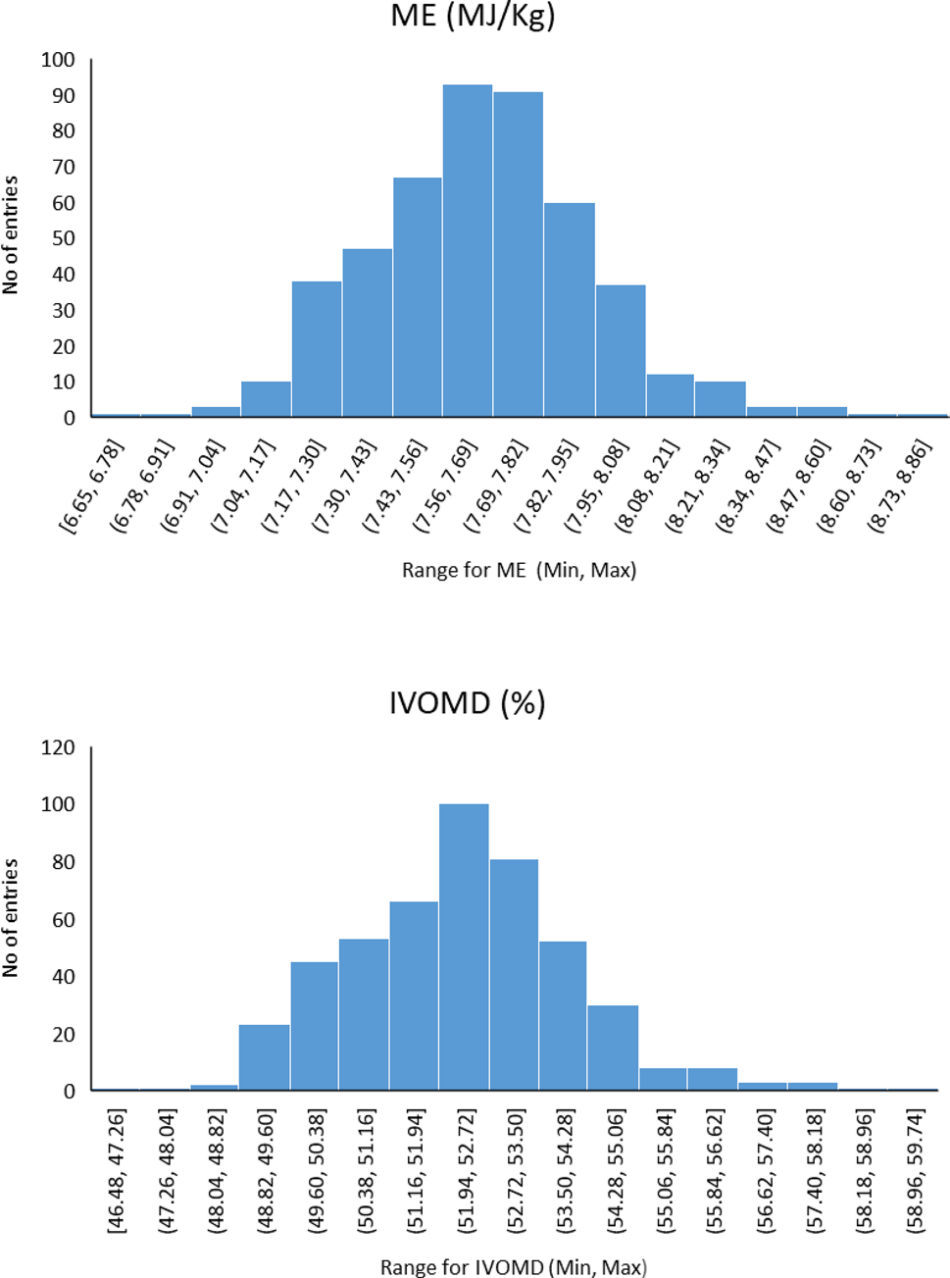
Frequency distribution for the two stover quality traits in association mapping panel and the training set.

Phenotypic distribution for the two traits (IVOMD and ME) in the panel was close to normal (Fig. 1a,b). Mean and ranges observed for the two stover quality traits is presented in Table 1. Significant variations were recorded among lines for both the traits across the panel (*P* = 0.006 for IVOMD and 0.007 for ME). IVOMD ranged from 46.50 to 59.30% with a mean of 52.32% and ME ranged from 6.65 to 8.75 Mj kg^−1^ with a mean of 7.60 Mj kg^−1^. Broad sense heritability for the entries ranged between 0.22 and 0.23 for the two traits.

**Table 1:** Descriptive statistics for stover quality traits (IVOMD and ME) across association mapping panel and testing set.

Parameters	Association mapping panel		Training set		Testing set (DH lines)	
	IVOMD (%)	ME(MJ/Kg)	IVOMD (%)	ME(MJ/Kg)	IVOMD (%)	ME(MJ/Kg)
Heritability	0.23	0.22	0.61	0.60	0.54	0.54
Genotype Variance	0.74	0.02	1.88	0.05	1.60	0.52
Grand mean	52.32	7.66	48.39	7.14	52.26	7.54
No of individuals	424	424	276	276	100	100
Range	46.50–59.30	6.65–8.75	42.50–53.91	6.25–8.04	50.02–54.88	7.19–7.91
Genotypic Significance (*P* value)	0.006	0.007	2.25E−06	3.56E−06	0.01	0.01

### Genome-wide association mapping

**Figure 2:**
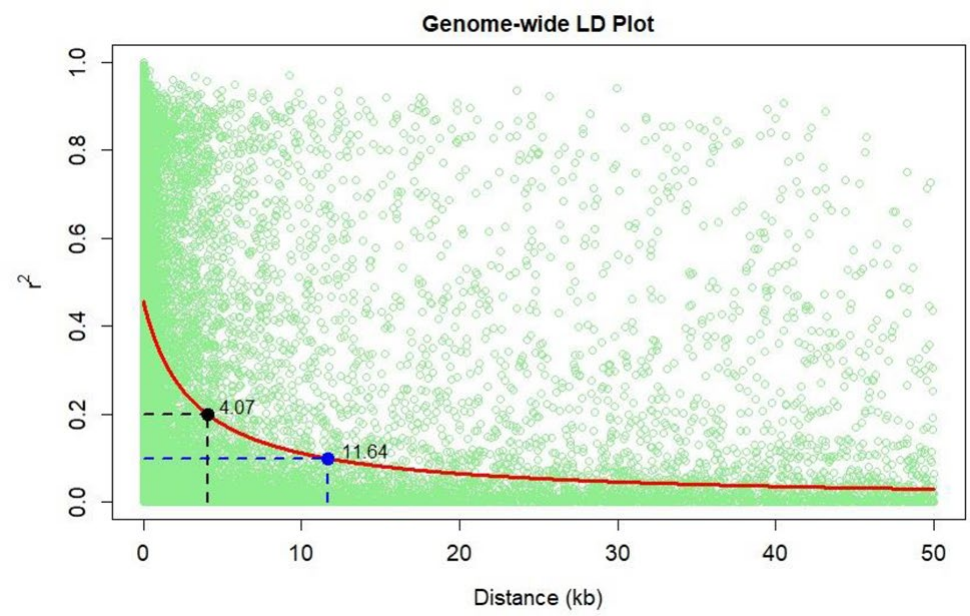
LD decay for the association mapping panel at r^2^ = 0.1 and 0.2.

**Figure 3:**
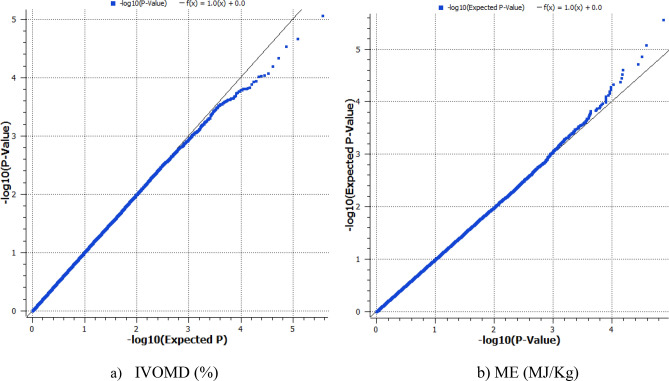
Quantile–quantile plots representing the SNP associations through MLM for the two stover quality traits (**a**) IVOMD and (**b**) ME.

**Figure 4:**
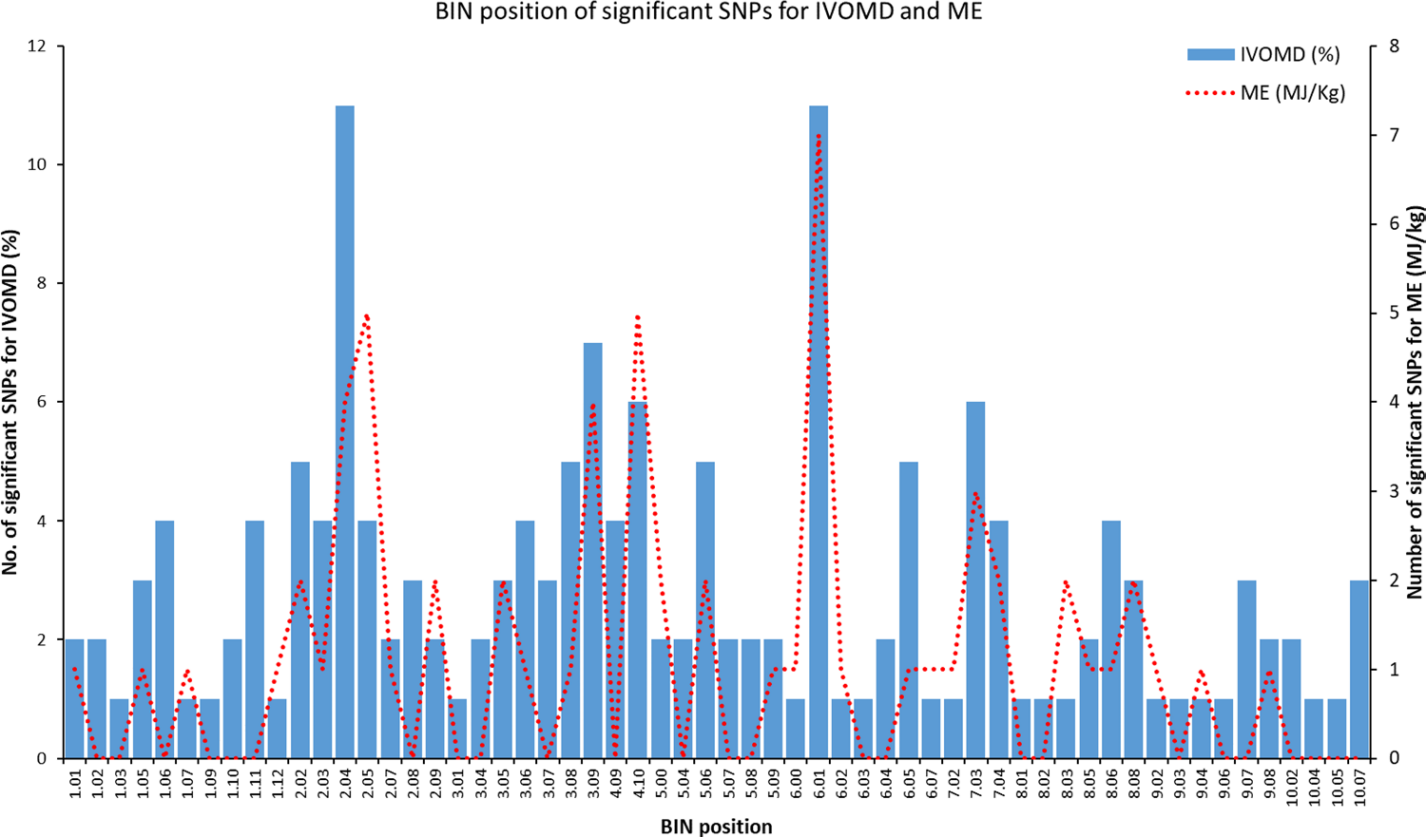
BIN positions for the identified significant SNPs for IVOMD (%) and ME (MJ/Kg).

**Figure 5:**
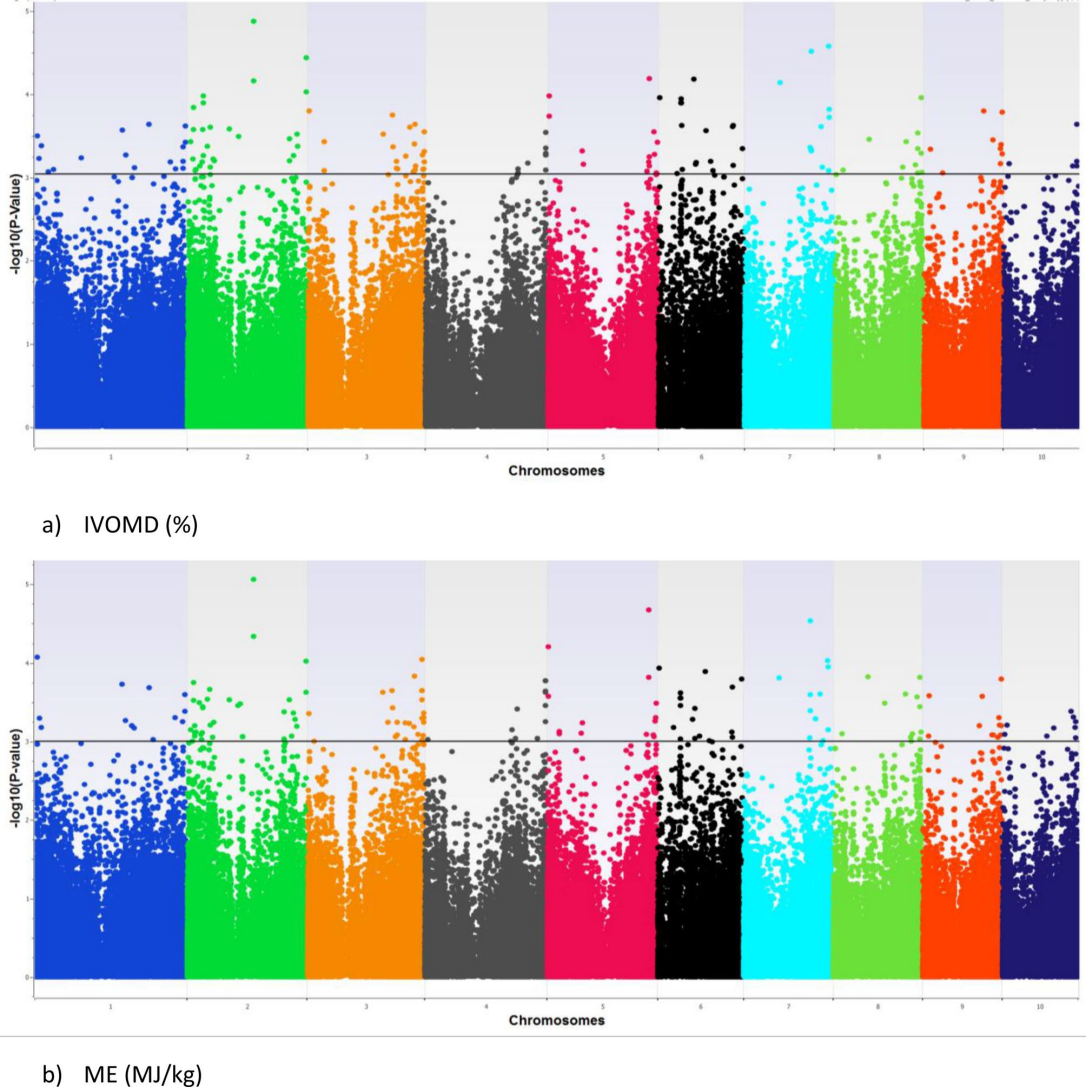
Manhattan plot depicting the association of SNPs for the two stover quality traits (**a**) IVOMD and (**b**) ME.

The population showed a high LD decay of 4.0 Kb at r^2^ of 0.2 and 11.6 Kb at r^2^ of 0.1 (Fig. 2). Identification of significant SNPs for IVOMD and ME was based on the Mixed linear model (MLM) fitted using population structure(Q) and relative kinship (K) as it had the best fit Q–Q plot (Fig. 3a,b). SNP associations identified were corrected for structure using ten principal components cumulatively accounting for close to 80% of the variation (Supplementary Fig. 1). For the study we used a threshold of *P* < 1 × 10^−4^, as a multiple testing correction like Bonferroni correction was too stringent for identifying any SNP association in this study. A large number of SNP associations at *P* value < 0.001 were observed for both the traits, however, the most significant associations (*P* value < 1 × 10^−4^) were detected amongst 9 SNPs for IVOMD and ME across the genome. Phenotypic variance explained by these individual SNPs identified for IVOMD ranged between 3.2 and 4.0% and the allelic effects ranged from -0.45 to 0.55. The phenotypic variance explained by these highly significant SNPs associated with ME ranged between 3.2 and 4.9% and the effects ranged from − 0.08 to 0.09 (Table 2). In addition, a lower threshold (*P* value < 1 × 10^−3^) was used for comparison of the SNPs from this study with the results with several previous studies on digestibility traits in maize (Supplementary Table 1a and b). A large proportion of significant SNP associations identified for ME in the current study were common or congruent (within 1 Mbp) to the associations identified for IVOMD. Among the identified SNPs with *P* value less than 1 × 10^−3^ (163 SNPs for IVOMD and 63 SNPs for ME), a large number of SNPs were found on chromosome 2, 3 and 6 for IVOMD and on chromosome 2 and 6 for ME (Supplementary Table 1a and b). These SNPs were clustered within one or two bins in each chromosome. The highest number of SNPs for IVOMD and ME were found on bins 2.04–2.05 and 6.01 (Fig. 4). SNPs with the lowest *P* value < 10^−5^ were found on chromosome 2 (bins 2.05 and 2.09), 5 (bin 5.06), 6 (bin 6.01) and 7 (bin 7.02–7.04) for IVOMD with *P* value < 9.3 × 10^−5^ and on chromosome 1 (bin1.01), 2 (bins 2.05 and 2.09), 3 (bin 3.09), 5 (bins 5.00 and 5.06) and 7 (bins 7.03 and 7.04) for ME with *P* value < 9.5 × 10^−5^. While significant SNPs were identified for IVOMD across the chromosomes (1–10), the study could not detect any significant SNPs for ME on chromosome 10 (Fig. 5a,b). These significant SNP associations and their allelic effects along with their physical position are detailed in Table 2.

**Table 2:** Top SNPs associated with in vitro organic matter digestibility (IVOMD) and metabolizable energy (ME).

Trait	Bin position	Marker	Chromosome	Physical position (Kbp)	*P* value	R^2^ (%)	Favorable Allele	Allele effect
IVOMD	2.05	S2_131425963	2	131,425,963	1.34E−05	4.0	C	− 0.327
ME					8.70E−06	4.2	C	− 0.054
IVOMD	2.05	S2_131425968	2	131,425,968	6.92E−05	3.3	G	− 0.294
ME					4.60E−05	3.5	G	− 0.049
IVOMD	2.09	S2_235226296	2	235,226,296	3.64E−05	3.6	C	− 0.446
ME					9.58E−05	3.2	C	− 0.070
IVOMD	5.06	S5_200435108	5	200,435,108	6.47E−05	3.4	A	− 0.297
ME					2.14E−05	3.8	A	− 0.053
IVOMD	7.03	S7_131438148	7	131,438,148	3.07E−05	3.7	A	0.554
ME					2.95E−05	3.7	A	0.090
IVOMD	7.04	S7_165798736	7	165,798,736	2.63E−05	3.7	T	− 0.372
ME					9.39E−05	3.2	T	− 0.061
IVOMD	6.01	S6_69394399	6	69,394,399	6.65E−05	3.4	A	0.376
	7.02	S7_69997119	7	69,997,119	7.25E−05	3.3	T	− 0.397
ME	1.01	S1_4724733	1	4,724,733	8.46E−05	3.3	G	0.050
	3.09	S3_227586235	3	227,586,235	9.13E−05	3.2	G	− 0.084
	5.00	S5_2400437	5	2,400,437	6.31E−05	3.4	A	− 0.054

### Genomic prediction for stover quality traits

The primary training set for the genomic prediction estimation, involved the phenotypes from a set of 276 elite breeding lines from the working germplasm of the program, and the test set comprised of DH lines derived from the elite breeding crosses of the program. After excluding the SNPs based on the filtering criteria across the genotypic datasets of training and test set, a total of 156,884 SNPs were used to estimate the genomic breeding values. In addition, the SNP dataset was further sub-divided into a varied number of marker densities (500, 1000, 3000, 50,000, 10,000, 50,000, 100,000) to determine the prediction accuracies at different marker densities. The sub-sets of the genotype for the different marker densities were made at least twenty times and the prediction accuracies averaged over these iterations. For comparison, genomic predictions were also estimated for the selected DH lines by using the association mapping panel set as a training set.

### Genomic prediction of test set and estimation of prediction accuracy

Test crosses of the 276 breeding lines used as the primary training set were phenotyped for both stover quality traits and exhibited substantial variation—IVOMD (47.60 to 53.91%) and ME (6.25 to 8.04 Mj kg^−1^) Table 1. The training set was genotyped at high density with the GBS platform. The resulting maker density, after following the filtering criterion across the dataset, was one SNP per 13.11 kb. IVOMD and ME of test population comprising of the DH lines were predicted based on the GBLUP prediction model developed on the training set. Predicted phenotype for IVOMD ranged from 47.22 to 49.27% with an average of 48.43% and for ME it ranged between 6.96 and 7.27 Mj kg^−1^ with a mean of 7.15 Mj kg^−1^ (Supplementary Table 2).

**Figure 6:**
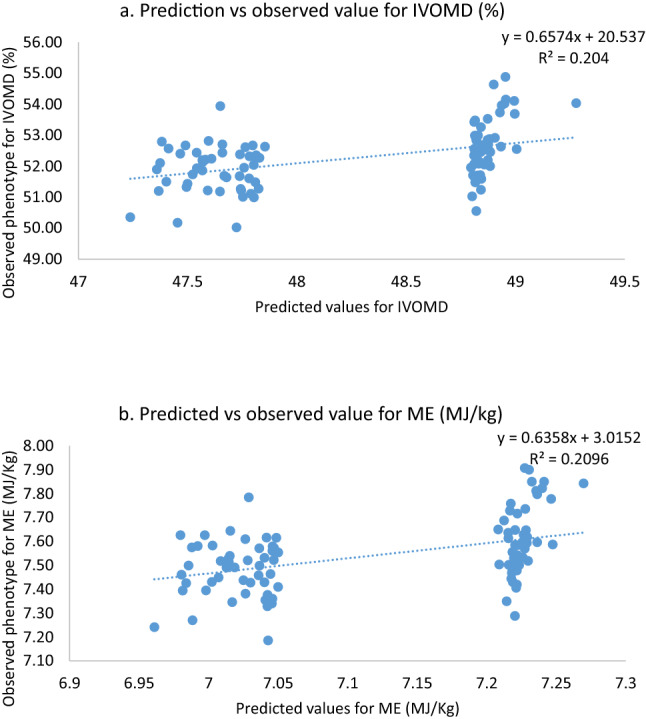
Graph representing predicted vs observed values among DH lines for the two stover quality traits (**a**) IVOMD and (**b**) ME.

To validate the prediction accuracy of the genomic selection, 100 DH lines were sampled from the test population based on the two extremes of the predicted values (top 5% and bottom 5%) for the stover traits, IVOMD and ME. The entries selected with in the top 5% matched across both the traits evaluated with an exception of only 4 entries with 2 entries each that had high IVOMD predictions but moderate ME predictions and vice versa. However, for all further estimations, these four entries were considered within the high phenotype groups for both IVOMD and ME. Based on these predictions, the set of 100 lines selected were test crossed and phenotyped for the two stover quality traits. The observed phenotypic value of IVOMD and ME ranged from 50.02 to 54.88% and 7.19 to 7.91 Mj kg^−1^, respectively with a broad-sense heritability of 0.54. Descriptive statistics of testing set is presented in Table 1. Prediction accuracy was estimated based on the Pearson’s correlation coefficient between predicted/estimated and observed phenotype across both high and low groups and also across the group for both the traits. Correlation coefficient between predicted and observed values for both the traits were high and significant across the group (r = 0.46 for IVOMD and ME) (Table 3). The prediction accuracies with in the high group of both the traits was substantially higher (r ~ 0.50), however this relationship was dismal within the low group of entries (Supplementary Table 3 and Fig. 6a,b), In addition, prediction accuracies at different marker densities selected at random, starting with low followed by higher densities were also estimated for both the traits across the groups, i.e. from a low marker density of 200 SNPs to a high marker density of > 100,000 SNPs. These results indicated that while prediction accuracies improved from low marker density to high density (0.42 to 0.46), they did not vary substantially at different densities and were largely similar (Table 3). In addition, the prediction was also done at different marker densities for the select 100 DH entries using the association mapping panel as a training set and also by combining the association panel and the primary training population (breeding lines) dataset. Based on the commonality with the testing set and filtering criterion a set of 127,623 SNPs were filtered out for the predictions. These marker datasets were further subdivided to varied marker density (200 to 127,623 SNPs) dataset for prediction. The prediction accuracy was high (r = 0.32 to 0.43) and comparable at different marker densities with training set comprising of both the breeding lines and entries from the association mapping panel (Table 3). These accuracies obtained were also comparable to that obtained when the training set comprised solely of the breeding panel. However, these accuracies were very low (r = 0.11 to 0.02) at all marker densities when the training set comprised of only entries in the association panel (Table 3).

**Table 3:** Prediction accuracy as determined by Pearson’s coefficient between predicted value estimated at different marker density (sampled 20 times) and the observed phenotype using the training set of advanced related breeding lines and the association mapping panel.

Marker density (SNPs)	Training set		Association mapping panel + training set		Association mapping panel	
	IVOMD (%)	ME (MJ/Kg)	IVOMD (%)	ME (MJ/Kg)	IVOMD (%)	ME (MJ/Kg)
200	0.36	0.42	0.33	0.32	− 0.11	− 0.10
500	0.42	0.43	0.36	0.39	− 0.12	− 0.14
1000	0.43	0.45	0.42	0.45	0.10	0.06
3000	0.44	0.46	0.40	0.40	− 0.09	− 0.13
5000	0.45	0.45	0.43	0.45	− 0.04	− 0.10
10,000	0.45	0.45	0.43	0.45	0.11	0.04
50,000	0.45	0.46	0.41	0.42	− 0.01	− 0.07
100,000	0.45	0.46	0.41	0.42	− 0.02	− 0.09
> 100,000^a^	0.45	0.46	0.41	0.43	− 0.02	− 0.09

^a^Sampled only one time with the total SNP markers (156,884 SNPs for testing set and 127,623 SNPs for Association mapping panel + Training set).

## Discussion

Sustainability for marginal and poor livestock holders can be achieved by ensuring the availability of feed throughout the year^4^. Crop residues of cereals—the by-products after harvest of the primary product (grains), are emerging as the major alternative to feed resources in developing countries where feed demand is steadily increasing while the availability of natural resources, particularly arable land and water, is declining^42^. Crop residues do not cost additional input such as water, land, etc., as crops are invariably grown for the harvest of grain^4^. Among the cereal crops, maize, which is grown throughout the year in tropical climate, has a very high demand and potential to be a successful dual-purpose crop. Most of the maize breeding programs of south Asia largely focus on improving grain yield—under optimal conditions with reduced losses under various biotic and abiotic stress conditions. However, global increase in demand for fodder has created an impetus for breeders to concentrate on ways of whole plant multiple trait improvement, concomitantly targeting food, feed and fodder improvement.

Considerable progress has been made in detecting and exploiting genetic variations in stover fodder quality in maize. Screening of many of the released and pipeline hybrids of maize for stover quality has resulted in the identification and preferential promotion of dual-purpose hybrids across the globe, including USA^43^, Mexico^44^, Ethiopia and Tanzania^9^, South Africa^45^ and South Asia^8,32,46^ However, targeted breeding for these dual purpose (grain and stover) maize would entail additional phenotyping and increase in cost of the breeding pipeline, a major bottleneck for whole plant improvement for multiple traits. Use of molecular markers circumventing the need for additional phenotyping could support breeders in early selections of genotypes with superior yields and stover quality. Genomic prediction/selection approach could also help breeders design population improvement schemes for simultaneous improvement of multiple traits and complete multiple selection cycles within the time period conventionally used for improving a single trait^47–49^. However, applicability of GS model for trait of interest and/or reliability of the results of an association mapping for integration in breeding pipeline needs a deeper assessment. Two stover quality traits IVOMD and ME were selected for this study, as they are considered two of the most desired traits of stover quality and a small improvement in these traits can positively impact animal productivity^50^. We reviewed the previous association mapping studies and identified a suite of associations that co-localize for both the traits and address their wider applicability and integration into the maize breeding pipeline. In addition, we attempted to assess the applicability of the genomic prediction model for these traits in the current breeding pipeline.

The set of entries used as the association mapping panel consisted largely of tropical germplasm from across South Asia. Linkage disequilibrium (LD) decay of this set was high (4.0 Kb at r^2^ of 0.2 and 11.6 Kb at r^2^ of 0.1) and was similar to that observed in previous studies on tropical germplasm^27,32,51^. The low LD decay as seen in the current study further suggests that the panel is diverse, and hence the use of higher number of SNPs should provide a good resolution. The current association study used 181,768 SNPs for the association mapping. The association tests in our study were corrected for both structure and kinship. First 10 principal components used to correct for structure in the current analysis explained close to 81% of the variation. Considering the bonferroni corrected P value was too stringent to identify significant SNPs, a lower threshold of *P* value < 1 × 10^−4^ was used as a cut off for identifying potential SNPs. A set of 64 SNPs for ME and 163SNPs for IVOMD were identified for both the traits (*P* value < 1 × 10^−3^) through single locus MLM approach.

The two stover quality traits are reported to be highly correlated^8,32,52^ and as expected most the identified putative SNPs in the current study for the two traits co-localized suggesting a common genetic control. Identifying genomic regions contributing to the traits could greatly increase their applicability and ease of integration in mainstream breeding. A large number of significant SNPs identified clustered on chromosome 2 (11 SNPs at bin 2.04) and chromosome 6 (11 SNPs at bin 6.01). The bin 2.04 has been identified previously to contain QTLs for several fodder quality traits in maize (IVOMD, NDF, ADF, ADL)^21–23,53–56^, while Meta QTLs and cell wall digestibility traits have also been reported on bin 6.01^23^. These regions have also been found to be associated with gene models contributing to proteins involved in various cell wall components. The gene model GRMZM2G479018 (bin 2.04) associated with a SNP (S2_39751613) was found to encode for ABC like transporters. These proteins are associated with transport of lignin precursors from cytoplasm through plasmalemma and sequestration in vacuoles in Arabidopsis^57^. Lignin an important component of plant stem cell wall reduces the enzymatic degradability of biomass and cell wall polysaccharides in the rumen^58,59^. The study also identified SNPs (S6_2043652 and S6_35895666) on bin 6.01 associated with gene model (GRMZM2G035741) encoding G protein beta subunit, WD 40 repeat and BRI1-KD interacting protein 130 (GRMZM2G035741) that phosphorylates G protein for sugar signaling. Role of G protein and WD 40 repeats in cell wall biogenesis has been reported in earlier studies in Arabidopsis^60,61^.

Another hot spot with clusters of SNP for IVOMD and ME was observed on chromosomes 3 (bin 3.09) and 4 (bin 4.1). QTL for cell wall composition and neutral sugar component of cell wall, glucose and ferrulic acid have been identified previously on bin 3.09^62^. A meta QTL for cell wall digestibility traits has also been reported^23^ on bin 4.1. This region was also identified to harbor QTLS for ADF and NDF^53^.The highest variation among the putative SNPs along with the lowest *P* value was accounted by SNPs on chromosome 2 (bin 2.05 and 2.09), chromosome 5 (bin 5.06) and chromosome 7 (bin 7.03 and 7.04) across both the traits IVOMD and ME. QTLs have been found for various cell wall component digestibility traits such as ADF, NDF and IVDMD on bin 2.05^56^ and meta QTL for cell wall traits was reported in this region^23^. QTLs for p-coumaric esters (PCA) involved in lignin biosynthesis have also been reported in this region. Meta QTL for cell wall component and digestibility traits have also been reported^23^ on bin 2.08–2.09, bin 5.06, bin 7.03 and 7.04. Bin 7.04 has also been found to harbor QTLs for cell wall digestibility traits ADF and NDF^53^. QTLs for cell wall component arabinose (7.03) and Klason lignin (bin 7.04) has also been reported previously^62^ on chromosome 7. Arabinoxylon is responsible for cross linking of lignin with ferulate bridges and is known to contribute to the reduced rumen degradability of maize cell walls^63,64^. In addition to these, three of the identified putative SNPs on chromosome 9 (bin 9.07) (S9_151819648, S9_151654313, S9_152760384) and one on chromosome 3 (bin 3.05) (S3_149247409) co-localized (within 1 Mb) to the identified SNP for IVOMD from the previous study^32^. These SNPs are co-localized to the regions coding for the enzymes protein phosphatase and urate hydroxylase, involved in signal transduction. This region on chromosome 9 (bin 9.07) has also been previously identified as a major hotspot region for cell wall components and digestibility in a meta-analysis study^23^. While several significant associations were detected in the current study, most of these associations for the traits observed had minor effects and were associated with low phenotypic variance. Previous studies have also identified a large number of minor effect QTLs for forage quality^23^ in maize restricting the use of SNPs in breeding programs. Further, the need of repeat phenotyping of such traits with low heritability can often unsettle the breeding pipeline and substantially increase the cost of the breeding program. Hence, genomic selection strategy might be the best possible approach for their rapid improvement.

Trait heritability, relationship between the training and testing set, variability for the trait of interest in the training population are few of the major factors determining success of genomic selection^41^. In the current study, the training set exhibited substantial variation (*P* < 0.0001) and good repeatability (h^2^ > 0.50) for the two traits, IVOMD and ME. In addition, as GWAS did not identify any major regions for deployment, the use of genomic prediction strategy for these traits might be an effective way of improving the trait. To understand the applicability of whole genome predictions for these traits, a set of 1026 DH lines derived from 10 bi-parental breeding crosses from the program were used as the prediction set. The genomic selection model was trained based on two different training sets (i) The first training set consisted of 276 test crosses of active breeding lines from the Asia program, that included the founders of the DH lines and (ii) the second training set consists of the association mapping panel used in the study comprising diverse inbred lines.

Based on the predicted trait values using the first training set consisting of the test crosses of the active breeding lines, a set of 100 inbred lines were selected (top and bottom ranking) and evaluated for the traits IVOMD and ME. The correlation between the observed phenotype of the selected lines of the testing population and the corresponding predicted high phenotype of those lines was significantly positive. The level of prediction accuracy in the present study was similar to or higher than the level for several agronomic and disease traits in other studies reported earlier^65–69^. Moreover, the success of prediction of traits through genome-wide genotyping depends on various features like marker density relative to the population’s effective size, persistence of LD in the breeding material, etc.^70–72^ and similarity of the training and test set. In the current study, the parental lines of the crosses used for developing the DH populations were also part of the breeding lines involved in the training set indicative of the close similarity of the training and test sets.

To determine if similar prediction accuracy could be achieved when the training set was unrelated to the test set, the association mapping panel, which is more like a diversity panel, was used as a training set to predict the performance of the selected DH lines. The results clearly showed poor prediction accuracies in this scenario, when there was no relation between the training and test set. However, the prediction accuracies improved when the training set constituted a combination of both the breeding lines and association panel. Further, the prediction accuracies were comparable to the results obtained when the training set comprised solely of the breeding lines. Relationship between test set and training set in a genomic prediction model affects genomic selection^73^. The highest prediction accuracies were found when training data represented the whole population and had a strong relationship to the test data^74^, while low prediction accuracies for genomic selection have been reported with less related individuals in the training and test population^75,76^. Besides relationship between training and test set, marker densities also affect in the prediction accuracies^39^. Considering these the prediction accuracies were also repeated with a suite of marker densities (200 to 100,000 SNPs) chosen at random from the total high density SNP markers. The average prediction accuracies as determined by Pearsons correlation coefficient across the different marker densities were comparable indicating that markers at lower densities could predict for these traits in a test set with accuracies similar to those obtained from higher densities across the three training sets. Zhang et al.^41^ found for less complex traits the increase in marker density could reduce the prediction accuracy, while for complex traits increased marker densities increased the prediction accuracy which stabilized after certain marker densities. However, in our study we did not find any significant differences in prediction accuracies at marker densities.

These preliminary leads in predicting fodder quality phenotype in bi-parental populations from prediction equations trained on an independent panel suggest that (i) the genomic selection approach could be effectively used to improve the two major stover quality traits in maize breeding populations; and (ii) that the approach could also be used as an effective tool of selecting parents for breeding starts for fodder quality trait improvement from the existing maize repositories without the need for extensive phenotyping. Further, good prediction accuracies for these traits even at low marker densities suggests that tailoring the current breeding program to integrate stover quality as a trait of interest might not be difficult.

## Conclusion

The study presented several significant SNPs for in vitro organic matter digestibility and metabolizable energy. The suite of SNPs identified co-localized to several QTLs previously reported, indicating their suitability for use in breeding programs. Hot spots with cluster of SNPs had been identified on chromosome 2 and 6 for both the traits, that would be interesting to further investigate. However, considering the traits are associated with several minor effect QTLs each accounting for low phenotypic variance, incorporating genomic selection in breeding programs in Asia to improve this trait seems to be the most optimum strategy. Good prediction accuracies between the training and testing sets for the two traits of interest even at a low density of markers as detailed in the study, further supports its use for improving stover quality.

## Materials and methods

### Germplasm

Three different sets of germplasm were used in generating experimental datasets in the current study, including an association mapping panel comprising 424 maize lines, a training set for genomic prediction that was constituted using a set of 276 Asia-adapted breeding lines from the working germplasm of the CIMMYT (International Maize and Wheat improvement Centre)—Asia breeding program, and a testing set for genomic prediction that comprised a set of untested 1026 double haploid (DH) lines derived from ten bi-parental breeding crosses that were genotyped and their breeding values estimated. A brief description of three set of germplasm is presented below.

#### Association mapping panel

An association mapping panel was constituted that comprised of 424 diverse maize inbred lines sourced from CIMMYT maize program (371 sub-tropical/tropical lines), Purdue University, USA (15 temperate lines) and the Maize and Millets Research Institute (MMRI), Pakistan (38 sub-tropical lines). The panel was test-crossed with a CIMMYT tester line with high general combining ability (CML451) and phenotyped for two key stover quality traits—in vitro organic matter digestibility (IVOMD) and metabolizable energy (ME) during post rainy season of 2013. This panel was also used as a secondary training set for predicting the performance of 100 DH lines selected for the two stover traits (IVOMD and ME).

#### Primary training set for genomic prediction

Phenotypic and genotypic datasets from 276 advanced stage maize inbred lines from the working germplasm of CIMMYT Asia maize breeding program were used as a primary training set in the genomic selection model for the two stover quality traits (IVOMD and ME). Lines involved in the study were Asia-adapted advanced elite breeding lines that originated from the sub-tropical and tropical maize breeding program of CIMMYT and being currently actively used as parental lines of several breeding crosses.

#### Testing set for genomic prediction

A set of 1026 double haploid (DH) lines derived from ten elite × elite bi-parental crosses were used as the testing set. The founder lines of these 10 DH populations were part of the primary training set used for prediction. These untested DH lines were genotyped using genotype-by-sequencing (GBS) platform and the breeding values for the two major stover quality traits were estimated using the model trained on the training set with > 150,000 random SNPs across the genome. Based on the genomic estimated breeding values (GEBVs), a sub-set of 100 DH lines was selected (50 high ranking and 50 low ranking) from the 1026 DH lines. Considering the two traits were highly correlated, there was little discrepancy in the selection, with only 4 entries not consistently falling under high IVOMD and high ME criterion. Two of these entries each had high IVOMD value but moderate ME values and vice versa. However, for the all estimation purposes these entries were considered in high groups. The selected 100 DH lines were planted as replicated trials and phenotyped for the two stover quality traits at Hyderabad during 2014 post Rainy season. Prediction accuracies were estimated at different marker densities based on the correlation coefficient between the predicted values and the observed phenotypic values on those 100 DH lines for the two stover quality traits.

### Experimental design

Test-cross progenies of association mapping panel and the primary training set were planted using alpha-lattice design with two replications during the spring seasons of 2013 respectively at the International Crops Research Institute for the Semi-Arid Tropics (ICRISAT) campus, Hyderabad, India. Each entry was planted in a 4-m long plot with a plant-to-plant spacing of 20 cm and a row-to-row spacing of 70 cm. Prior to planting, 60 kg nitrogen (N) ha^−1^ in the form of urea, 60 kg phosphorus ha^−1^ as single super phosphate, 40 kg potassium ha^−1^ as muriate of potash and 10 kg zinc as zinc sulfate were applied as a basal dose. The second and third doses of N (each 30 kg ha^−1^) were side-dressed when plants were at knee-high and at tasseling stage, respectively. Pre-emergence application of pendimethalin and atrazine [both at 0.75 kg ha^−1^ active ingredient (*a.i*.)*,* tank mixed] were used to keep the crop weed-free at early growth stages. Standard agronomic and plant protection measures were followed throughout the cropping period to prevent from biotic or abiotic stress affecting the stover quality. At the maturity stage after harvesting of the cobs, five representative plants were selected from each plot for stover quality analysis.

Based on the predicted values (GEBVs) for the two stover quality traits, a subset of 100 DH lines was selected out of 1026 DH lines. Test-crosses of these 100 DH lines were then planted in two replications at ICRISAT campus, Hyderabad, India following a similar design as detailed earlier during post Rainy season of 2014. The recommended agronomic practices were followed for a good crop stand. At maturity stage, five representative plants per plot were harvested for stover quality analysis.

### Phenotyping for stover fodder quality traits

Five whole plants were sampled from each of the plot in the experimental trials and processed for the various stover quality traits. The analysis for IVOMD and ME was done at the Livestock Nutritional Laboratory, International Livestock Research Institute (ILRI) at ICRISAT campus using Near Infrared Spectroscopy (NIRS), calibrated against conventional laboratory analyses. The NIRS instruments used were FOSS Forage Analyzer 5000, 6500 and XDS with the software package WinISI II. The procedure for analysis of maize stover samples for these quality traits using NIRS has been described in detail in Vinayan et al.^32^.

### Genotyping

All the three set of germplasm (AM panel for GWAS, training set of 276 elite lines and 1026 DH lines from biparental crosses as prediction set) used in the current study were genotyped using the GBS platform^77^ at the Institute for Genomic Diversity, Cornell University, Ithaca, NY, USA. A total of 955,690 SNPs were generated through GBS v2.7 and the SNPs were filtered for the analysis, based on the method described by Suwarno et al.^78^ with slight modifications. A call rate of > 0.85 and minor allelic frequency (MAF) > 0.05 criteria resulted in a set of 181,768 SNPs for association mapping analysis. Marker density for association mapping panel was one SNP per 11.604 kb. Structure of the association panel was determined using principal components, wherein the SNPs were further filtered based on a call rate of 0.9 and MAF < 0.1. In addition, Linkage Disequilibrium (LD) pruning based on adjacent markers was also carried out. This criterion resulted in a set of 55,807 SNP markers for principal component analysis.

To train the genomic model for the two traits, the genotypic dataset from both training set (276 breeding lines) and a prediction set (1026 DH lines) were combined and later filtered using a call rate of 0.9 and MAF of 0.1. This filtering criterion resulted in a set of 156,884 SNPs, which were then used to predict phenotypes of 1026 DH lines derived from 10 bi-parental breeding crosses, based on the marker effects obtained from the training sets. The study also used the association mapping panel as a secondary training set and the testing set comprised of 100 DH lines for estimating the prediction accuracies. For this purpose, the filtered genotyping dataset estimated from the primary training set, association mapping panel and DH lines were merged. Based on the commonality a final set of 127,623 markers were then used for the prediction. In addition, to the whole set of random markers (156,884) used for prediction, SNP marker densities of 200, 500, 1000, 3000, 5000, 10,000, 50,000 and 100,000 SNPs were also used to estimate the breeding value of the 100 DH lines.

### Statistical analysis of phenotypic data

A linear mixed model was used for the analysis of the stover quality traits using the web handle META-R developed by CIMMYT^79^,

$${Y}_{ijk}=\mu +{R}_{i}+{B}_{j}\left({R}_{i}\right)+{G}_{k}+{\varepsilon }_{ijk}$$

where ${Y}_{ijk}$ is the trait of interest, $\mu$ is the mean effect, ${R }_{i}$ is the effect of the ith replicate, ${{B}_{j}(R}_{i})$ is the effect of the jth incomplete block within the ith replicate, ${G}_{k}$ is the effect of the kth genotype and ${\varepsilon }_{ijk}$ is the error associated with the ith replication, jth incomplete block and the kth genotype. The effects of block, replicate and genotype were considered random to estimate the best linear unbiased predictors (BLUPs) which were used for the analysis. Single location heritability of the trials was computed using the genotypic variance estimates (${\sigma }^{2}g)$ and single location residual (${\sigma }^{2}\varepsilon )$ as

$${\omega }^{2}= {\sigma }^{2}g\left[ {\sigma }^{2}g + {\sigma }^{2}\varepsilon \right].$$

### Statistical analysis of genomic prediction and association analysis

Association tests in between markers and association mapping panel phenotypes were corrected for population structure and kinship following a single locus mixed linear model (MLM) (G + Q + K) following the efficient mixed-model association expedited (EMMAX) variance component approach^80^. The additive model was used for the analysis and the missing genotype values were set to 0. The kinship matrix used as covariates were treated as random effects. Manhattan plots were plotted using the − log 10 P values of all SNPs in the model and Quantile–quantile plots were plotted using the observed − log 10 P values and the expected − log 10 P values. A suite of significant SNP associations was observed from the analysis. These significant associations were then used to look for congruence with results from previous studies. The associated SNPs were considered co-localized if found to be in close proximity (within 1 Mbp) to the previously reported SNP for the trait^81^. The analysis were carried out using SNP and Variation Suites v8.6.0^82^. In addition, the gene models with the putative SNP associations were identified from maize GDB genome browser at http://www.maizegdb.org (B73 RefGen_V2) and their respective protein were obtained from Uniprot genome browser at http://uniprot.org.

Genomic predictions were done following genomic best linear unbiased prediction (G-BLUP) method^83^. The primary training set comprised of phenotypic and genotypic datasets of 276 CIMMYT Asia-based breeding lines, and the prediction set comprised of 1026 DH lines derived from 10 bi-parental crosses involving Asia-adapted elite breeding lines. The GBLUP model used here was

$$Y=\mu +g+e$$

where Y represents the trait IVOMD and ME, µ is the model intercept, g represents the vector of the genotypic values for the genotype and e is the residual. The GBLUP method computes a genomic relationship matrix and from that computes the “Genomic Best Linear Unbiased Predictor” (GBLUP) of additive genetic merits by sample. The genomic relationship matrix G is estimated as

$$G =\frac{WW{^\prime}}{2\sum pi(1-pi)},$$

where W represents the centered genotype matrix and pi represents the allele frequencies. The genotypic relationship matrix was estimated for each marker density and missing genotype data was recorded as 0 (the major homozygous allele). The Expectation–Maximization (EM) and Efficient Mixed Model Association (EMMA) algorithm was used for predicting the performance of the unpredicted lines. All the predictions were done on SNP and Variation Suites v8.6.0^82^. Based on the GEBVs of the DH lines estimated using the full set of 156,884 SNPs, a sub-set of 100 DH lines were subsampled. The test crosses form these lines were evaluated for the two traits to obtain the observed phenotypic value. The prediction accuracies were estimated as simple Pearson’s correlation coefficient between estimated breeding values and observed breeding values. Further, the prediction accuracies were re-estimated for these selected 100 DH lines at different marker densities. In addition, for comparison of prediction accuracies when a diversity panel is used in the breeding scheme, predicted values for these 100 DH lines were also estimated using the association mapping panel as the training set. The predicted values for DH lines were estimated by sampling genotype dataset at designated marker density 20 times. The correlation coefficients across these 20 samples for each marker density were then averaged following Fisher Z transformation as described by Alexander et al.^84^.

## Supplementary Information

Supplementary Information.
