# Supplementary material for: Genome wide association study and genomic prediction for stover quality traits in tropical maize (Zea mays L.)
Source: Sci Rep. 2021 Jan 12;11:686. doi: 10.1038/s41598-020-80118-2 (PMC7804097; doi:10.1038/s41598-020-80118-2)
Supplement: Supplementary file 1 — Supplementary Information. [file 41598_2020_80118_MOESM1_ESM.pdf]

Genome wide association study and genomic prediction for stover quality traits in tropical maize (*Zea mays* L.)

Vinayan M.T. [1,*], K. Seetharam[1], Raman Babu[1,#], P.H. Zaidi[1], M. Blummel[2], and Sudha K Nair[1]

Supplementary table 1a. Significant SNP associations for trait IVOMD through MLM

Marker	Chr.	bin	Position (bp)	Gene Model	P-Value	Proportion of Variance Explained	Minor Allele (Test Allele)	Minor Allele Frequency	Allele effect
S1_4724733	1	1.01	4724733	GRMZM2G105436_T01	3.15E-04	0.027	G	0.292	0.281
S1_8113222	1	1.01	8113222	GRMZM2G475059_T01, GRMZM2G475059_T02	5.89E-04	0.025	A	0.387	0.241
S1_12727343	1	1.02	12727343		4.16E-04	0.026	T	0.150	0.482
S1_27401555	1	1.02	27401555	AC197545.3_FGT002	8.52E-04	0.024	C	0.246	-0.210
S1_36832408	1	1.03	36832408	GRMZM2G072492_T01	8.04E-04	0.024	A	0.182	-0.353
S1_92465338	1	1.05	92465338	GRMZM2G094639_T01	5.81E-04	0.025	T	0.161	-0.255
S1_157239188	1	1.05	157239188		9.83E-04	0.023	G	0.295	-0.230
S1_173234207	1	1.05	173234207	GRMZM2G003509_T01, GRMZM2G003509_T02	2.69E-04	0.028	G	0.496	-0.243
S1_179925407	1	1.06	179925407	AC212323.4_FGT002	5.38E-04	0.025	G	0.227	-0.325
S1_192402808	1	1.06	192402808	GRMZM2G096585_T03, GRMZM2G096585_T02, GRMZM2G096585_T01, GRMZM2G096585_T04, GRMZM2G096585_T05, GRMZM2G096585_T06	9.99E-04	0.023	A	0.104	0.408
S1_192402818	1	1.06	192402818	GRMZM2G096585_T03, GRMZM2G096585_T02, GRMZM2G096585_T01, GRMZM2G096585_T04, GRMZM2G096585_T05, GRMZM2G096585_T06	9.99E-04	0.023	A	0.104	0.408
S1_197059006	1	1.06	197059006	GRMZM2G175280_T01	7.62E-04	0.024	G	0.297	-0.391
S1_226456940	1	1.07	226456940	GRMZM2G131817_T01, GRMZM2G131817_T02	2.29E-04	0.029	C	0.248	0.296

Marker	Chr.	bin	Position (bp)	Gene Model	P-Value	Proportion of Variance Explained	Minor Allele (Test Allele)	Minor Allele Frequency	Allele effect
S1_255835560	1	1.09	255835560	GRMZM2G026346_T02, GRMZM2G026346_T01	9.82E-04	0.023	G	0.202	-0.386
S1_268880779	1	1.1	268880779	GRMZM2G044569_T01, GRMZM2G044569_T02, GRMZM2G044569_T03, GRMZM2G044569_T04, GRMZM2G044569_T06	6.51E-04	0.025	T	0.281	-0.321
S1_278665426	1	1.1	278665426	GRMZM2G014955_T01	7.91E-04	0.024	A	0.116	0.591
S1_293629690	1	1.11	293629690	GRMZM2G368886_T02, GRMZM2G368886_T01	7.85E-04	0.024	A	0.145	0.470
S1_294085083	1	1.11	294085083		4.33E-04	0.026	G	0.493	-0.242
S1_294135460	1	1.11	294135460	GRMZM2G045781_T01, GRMZM2G045781_T02, GRMZM2G045781_T03	6.42E-04	0.025	A	0.159	0.370
S1_297861352	1	1.11	297861352		2.43E-04	0.029	T	0.063	0.432
S1_298273257	1	1.12	298273257	GRMZM2G159353_T01	3.78E-04	0.027	T	0.103	-0.513
S2_6333612	2	2.02	6333612		3.73E-04	0.027	A	0.257	-0.308
S2_10337822	2	2.02	10337822	GRMZM2G005310_T01, GRMZM2G005310_T02	6.07E-04	0.025	T	0.359	0.180
S2_12243893	2	2.02	12243893		2.68E-04	0.028	C	0.450	-0.232
S2_12243894	2	2.02	12243894		1.45E-04	0.031	G	0.348	-0.267
S2_14805724	2	2.02	14805724	GRMZM2G076771_T01	8.33E-04	0.024	A	0.134	-0.490
S2_16870117	2	2.03	16870117		8.08E-04	0.024	A	0.262	0.285
S2_24450868	2	2.03	24450868	GRMZM2G064124_T01	7.34E-04	0.024	A	0.076	0.496
S2_27581494	2	2.03	27581494		9.83E-04	0.023	T	0.442	-0.338
S2_28726272	2	2.03	28726272		6.57E-04	0.025	A	0.194	-0.307
S2_29834342	2	2.04	29834342		2.66E-04	0.028	G	0.266	-0.363
S2_30907772	2	2.04	30907772	GRMZM2G352129_T01	1.04E-04	0.032	C	0.251	-0.338
S2_30909931	2	2.04	30909931	GRMZM2G352129_T01	5.44E-04	0.025	G	0.313	-0.314
S2_30909942	2	2.04	30909942	GRMZM2G352129_T01	1.27E-04	0.031	T	0.229	-0.338
S2_30910013	2	2.04	30910013	GRMZM2G352129_T01	9.88E-04	0.023	C	0.229	-0.304
S2_39751613	2	2.04	39751613	GRMZM2G479018_T01	4.24E-04	0.026	C	0.082	-0.463

Marker	Chr.	bin	Position (bp)	Gene Model	P-Value	Proportion of Variance Explained	Minor Allele (Test Allele)	Minor Allele Frequency	Allele effect
S2_44721856	2	2.04	44721856	GRMZM2G355906_T02, GRMZM2G355906_T01, GRMZM2G355906_T04, GRMZM2G355906_T03, GRMZM5G834459_T01	2.48E-04	0.028	A	0.054	-0.619
S2_44721885	2	2.04	44721885	GRMZM2G355906_T02, GRMZM2G355906_T01, GRMZM2G355906_T04, GRMZM2G355906_T03, GRMZM5G834459_T01	7.59E-04	0.024	C	0.136	-0.496
S2_45603676	2	2.04	45603676	GRMZM2G441748_T01, GRMZM2G143891_T01	9.33E-04	0.023	T	0.060	-0.491
S2_50808880	2	2.04	50808880	GRMZM2G155626_T01	6.01E-04	0.025	T	0.068	-0.588
S2_52834504	2	2.04	52834504	GRMZM2G541230_T01	6.27E-04	0.025	T	0.063	-0.611
S2_83268098	2	2.05	83268098	GRMZM2G177575_T02, GRMZM2G177575_T03, GRMZM2G177575_T01, GRMZM2G177575_T04	2.61E-04	0.028	T	0.080	-0.638
S2_100778676	2	2.05	100778676		3.23E-04	0.027	A	0.070	0.633
S2_131425963	2	2.05	131425963	AC211746.3_FGT006	1.34E-05	0.040	C	0.291	-0.327
S2_131425968	2	2.05	131425968	AC211746.3_FGT006	6.92E-05	0.033	G	0.295	-0.294
S2_201966646	2	2.07	201966646	GRMZM2G113794_T01, GRMZM5G858373_T01	3.42E-04	0.027	A	0.209	0.337

Marker	Chr.	bin	Position (bp)	Gene Model	P-Value	Proportion of Variance Explained	Minor Allele (Test Allele)	Minor Allele Frequency	Allele effect
S2_202275566	2	2.07	202275566	GRMZM2G179505_T06, GRMZM2G179505_T04, GRMZM2G179505_T05, GRMZM2G179505_T01, GRMZM2G179505_T02	6.32E-04	0.025	C	0.077	0.692
S2_210393678	2	2.08	210393678	GRMZM2G180950_T01	5.47E-04	0.025	C	0.065	0.607
S2_217097445	2	2.08	217097445	GRMZM2G030646_T01	3.01E-04	0.028	A	0.405	-0.379
S2_218460160	2	2.08	218460160	GRMZM2G129209_T01	4.24E-04	0.026	G	0.261	0.371
S2_235226296	2	2.09	235226296	GRMZM2G178136_T01	3.64E-05	0.036	C	0.307	-0.446
S2_235226332	2	2.09	235226332	GRMZM2G178136_T01	9.36E-05	0.032	C	0.298	-0.441
S3_3112002	3	3.01	3112002	GRMZM2G062076_T01, GRMZM2G062076_T02	1.60E-04	0.030	C	0.181	0.476
S3_32033758	3	3.04	32033758	GRMZM2G049895_T01, GRMZM2G049895_T02	8.31E-04	0.024	G	0.479	0.135
S3_32033817	3	3.04	32033817	GRMZM2G049895_T01, GRMZM2G049895_T02	3.72E-04	0.027	A	0.314	-0.241
S3_149247409	3	3.05	149247409	GRMZM5G833593_T03, GRMZM5G833593_T04, GRMZM5G833593_T05, GRMZM5G833593_T01, GRMZM5G833593_T02	3.01E-04	0.028	T	0.158	0.323
S3_159817712	3	3.05	159817712	GRMZM2G133428_T02, GRMZM2G133428_T01, GRMZM2G133483_T01	9.26E-04	0.023	T	0.324	-0.247
S3_168012590	3	3.05	168012590	GRMZM2G392710_T01, GRMZM2G392710_T02	1.79E-04	0.030	C	0.396	-0.219

Marker	Chr.	bin	Position (bp)	Gene Model	P-Value	Proportion of Variance Explained	Minor Allele (Test Allele)	Minor Allele Frequency	Allele effect
S3_168838080	3	3.06	168838080	GRMZM2G156861_T03, GRMZM2G156861_T01, GRMZM2G458554_T01	4.29E-04	0.026	A	0.062	0.502
S3_175163368	3	3.06	175163368		7.80E-04	0.024	A	0.351	0.211
S3_175163403	3	3.06	175163403		6.45E-04	0.025	T	0.352	0.217
S3_178154505	3	3.06	178154505		7.96E-04	0.024	T	0.275	0.291
S3_194011353	3	3.07	194011353	GRMZM2G320013_T01	5.55E-04	0.025	G	0.411	-0.161
S3_194011374	3	3.07	194011374	GRMZM2G320013_T01	9.60E-04	0.023	A	0.483	-0.138
S3_201625485	3	3.07	201625485	GRMZM2G025860_T01	2.49E-04	0.028	A	0.098	0.572
S3_208969852	3	3.08	208969852	GRMZM2G423193_T01, GRMZM2G423202_T01	8.58E-04	0.024	T	0.446	0.462
S3_211531740	3	3.08	211531740		3.98E-04	0.027	A	0.088	0.551
S3_212406341	3	3.08	212406341	GRMZM5G822563_T03, GRMZM5G822563_T02, GRMZM5G822563_T01	2.32E-04	0.029	T	0.071	-0.462
S3_212415853	3	3.08	212415853	GRMZM5G822563_T03, GRMZM5G822563_T02, GRMZM5G822563_T01	8.23E-04	0.024	G	0.056	-0.480
S3_213559820	3	3.08	213559820	GRMZM2G022311_T01	7.33E-04	0.024	A	0.479	0.103
S3_227586235	3	3.09	227586235	GRMZM2G035785_T01	5.39E-04	0.025	G	0.176	-0.455
S3_228449827	3	3.09	228449827	GRMZM2G409312_T01, GRMZM5G862442_T01	8.05E-04	0.024	T	0.321	-0.203
S3_229253208	3	3.09	229253208	GRMZM2G002999_T01	9.67E-04	0.023	T	0.128	-0.253
S3_229678492	3	3.09	229678492	GRMZM2G109618_T03, GRMZM2G109618_T02, GRMZM2G109618_T01	7.90E-04	0.024	T	0.373	0.206
S3_230442595	3	3.09	230442595	GRMZM2G353103_T01	4.89E-04	0.026	A	0.098	-0.396
S3_230442601	3	3.09	230442601	GRMZM2G353103_T01	2.84E-04	0.028	C	0.214	-0.216
S3_230442640	3	3.09	230442640	GRMZM2G353103_T01	6.56E-04	0.025	A	0.100	-0.366

Marker	Chr.	bin	Position (bp)	Gene Model	P-Value	Proportion of Variance Explained	Minor Allele (Test Allele)	Minor Allele Frequency	Allele effect
S4_181889201	4	4.08	181889201	GRMZM2G041959_T01, GRMZM2G041959_T03, GRMZM2G041959_T05, GRMZM2G041959_T04	9.33E-04	0.023	T	0.322	0.267
S4_182947054	4	4.08	182947054	GRMZM2G011469_T01, GRMZM2G011469_T02	8.90E-04	0.023	T	0.069	-0.303
S4_183405219	4	4.08	183405219	GRMZM2G111720_T01	7.90E-04	0.024	T	0.352	0.240
S4_202279376	4	4.08	202279376	GRMZM2G100423_T01	6.69E-04	0.025	G	0.106	0.454
S4_238275507	4	4.1	238275507		5.38E-04	0.025	C	0.261	-0.417
S4_238275508	4	4.1	238275508		5.38E-04	0.025	A	0.261	-0.415
S4_238275759	4	4.1	238275759		5.10E-04	0.026	T	0.479	-0.309
S4_238275784	4	4.1	238275784		4.47E-04	0.026	G	0.492	-0.315
S4_238275785	4	4.1	238275785		8.19E-04	0.024	A	0.482	-0.300
S4_238357174	4	4.1	238357174	GRMZM2G423137_T02, GRMZM2G423137_T01, GRMZM5G878640_T02, GRMZM5G878640_T01	2.90E-04	0.028	A	0.170	0.429
S5_2188003	5	5	2188003	GRMZM2G085336_T01, GRMZM2G085336_T02	1.84E-04	0.030	G	0.402	-0.362
S5_2400437	5	5	2400437	GRMZM2G002765_T04, GRMZM2G002765_T02, GRMZM2G002765_T03, GRMZM2G002765_T01, GRMZM5G860816_T01	1.05E-04	0.032	A	0.391	-0.314
S5_67509001	5	5.03	67509001	GRMZM2G089836_T01, GRMZM2G089836_T02	4.82E-04	0.026	C	0.167	-0.393
S5_69456424	5	5.03	69456424	GRMZM5G854880_T01	6.92E-04	0.024	C	0.431	0.288
S5_196142106	5	5.06	196142106	GRMZM2G045977_T01, GRMZM2G045977_T02	8.38E-04	0.024	G	0.390	-0.189
S5_199230694	5	5.06	199230694		6.54E-04	0.025	C	0.339	-0.282

Marker	Chr.	bin	Position (bp)	Gene Model	P-Value	Proportion of Variance Explained	Minor Allele (Test Allele)	Minor Allele Frequency	Allele effect
S5_199984828	5	5.06	199984828		5.62E-04	0.025	T	0.065	-0.609
S5_200435108	5	5.06	200435108	GRMZM2G325019_T01	6.47E-05	0.034	A	0.308	-0.297
S5_200437468	5	5.06	200437468	GRMZM2G325019_T01	7.28E-04	0.024	C	0.372	-0.270
S5_209722095	5	5.07	209722095	GRMZM2G101613_T01	2.84E-04	0.028	G	0.499	-0.236
S5_210747392	5	5.07	210747392	GRMZM2G104920_T03	5.26E-04	0.025	A	0.061	-0.570
S5_213915805	5	5.08	213915805	GRMZM2G469142_T01, AC191361.4_FGT004	8.90E-04	0.023	T	0.059	-0.498
S5_214287165	5	5.08	214287165	GRMZM2G178887_T01	9.32E-04	0.023	C	0.485	-0.294
S5_215020212	5	5.08	215020212	GRMZM2G150932_T01, GRMZM2G150932_T02	9.04E-04	0.023	A	0.409	0.289
S5_215984446	5	5.09	215984446	GRMZM2G364172_T03, GRMZM2G364172_T02	3.79E-04	0.027	C	0.317	-0.183
S6_2043652	6	6	2043652	GRMZM2G009627_T01	1.10E-04	0.032	C	0.311	-0.250
S6_35895666	6	6.01	35895666	GRMZM2G035741_T02, GRMZM2G035741_T03, GRMZM2G035741_T01, GRMZM2G035741_T05	8.94E-04	0.023	C	0.346	0.307
S6_44821882	6	6.01	44821882		1.15E-04	0.031	C	0.395	-0.303
S6_44821884	6	6.01	44821884		1.28E-04	0.031	G	0.396	-0.301
S6_44821885	6	6.01	44821885		1.28E-04	0.031	C	0.396	-0.301
S6_44821886	6	6.01	44821886		1.28E-04	0.031	C	0.396	-0.301
S6_44821933	6	6.01	44821933		1.28E-04	0.031	C	0.396	-0.301
S6_45183186	6	6.01	45183186	GRMZM2G124617_T02, GRMZM2G124617_T01	2.38E-04	0.029	G	0.380	-0.306
S6_46776160	6	6.01	46776160	GRMZM2G008202_T02, GRMZM2G008202_T03, GRMZM2G008202_T01, GRMZM5G860077_T01	8.09E-04	0.024	G	0.251	0.229
S6_69394399	6	6.01	69394399	GRMZM2G172122_T01	6.65E-05	0.034	A	0.417	0.376
S6_73507199	6	6.01	73507199		6.95E-04	0.024	T	0.459	0.190
S6_73801877	6	6.01	73801877	AC209946.4_FGT002	6.67E-04	0.025	T	0.473	-0.262
S6_93495717	6	6.02	93495717	GRMZM2G161666_T01	2.72E-04	0.028	C	0.179	0.408
S6_102597111	6	6.03	102597111	GRMZM2G043310_T01	6.47E-04	0.025	C	0.104	0.660

Marker	Chr.	bin	Position (bp)	Gene Model	P-Value	Proportion of Variance Explained	Minor Allele (Test Allele)	Minor Allele Frequency	Allele effect
S6_107882211	6	6.04	107882211	GRMZM2G392700_T02, GRMZM2G392700_T01	8.35E-04	0.024	T	0.095	-0.540
S6_111022510	6	6.04	111022510	GRMZM2G138842_T01	9.47E-04	0.023	T	0.459	-0.297
S6_129471473	6	6.05	129471473	GRMZM2G131749_T01	9.93E-04	0.023	T	0.236	0.305
S6_129471492	6	6.05	129471492	GRMZM2G131749_T01	9.93E-04	0.023	G	0.236	0.305
S6_146218985	6	6.05	146218985	GRMZM2G080848_T01	2.46E-04	0.028	C	0.499	-0.236
S6_147868122	6	6.05	147868122		7.20E-04	0.024	G	0.272	-0.340
S6_147880140	6	6.05	147880140	GRMZM2G018971_T01	2.39E-04	0.029	T	0.470	0.290
S6_165945806	6	6.07	165945806	GRMZM2G520927_T01, GRMZM2G062738_T01	4.52E-04	0.026	C	0.440	-0.231
S7_69997119	7	7.02	69997119		7.25E-05	0.033	T	0.159	-0.397
S7_129344449	7	7.03	129344449	GRMZM2G115304_T01, GRMZM2G115304_T02	4.38E-04	0.026	A	0.388	-0.313
S7_131438148	7	7.03	131438148	GRMZM2G018108_T01, GRMZM2G018108_T02	3.07E-05	0.037	A	0.130	0.554
S7_132107595	7	7.03	132107595	GRMZM2G478160_T01	4.85E-04	0.026	A	0.251	0.452
S7_132169495	7	7.03	132169495		4.63E-04	0.026	G	0.104	0.596
S7_151404619	7	7.03	151404619		2.46E-04	0.028	C	0.215	-0.306
S7_153655433	7	7.03	153655433	GRMZM2G167591_T01, GRMZM2G167591_T02	7.51E-04	0.024	G	0.397	0.241
S7_165798736	7	7.04	165798736		2.63E-05	0.037	T	0.217	-0.372
S7_165798738	7	7.04	165798738		8.31E-04	0.024	C	0.208	-0.307
S7_167348106	7	7.04	167348106	GRMZM2G158130_T01	1.90E-04	0.029	A	0.101	0.493
S7_167634171	7	7.04	167634171		1.52E-04	0.030	T	0.257	-0.337
S8_3662555	8	8.01	3662555	GRMZM2G074857_T01, GRMZM2G074857_T02	9.33E-04	0.023	T	0.386	-0.212
S8_16557289	8	8.02	16557289	GRMZM2G175334_T01	8.16E-04	0.024	T	0.132	0.605
S8_68087278	8	8.03	68087278		3.47E-04	0.027	A	0.061	0.634
S8_135452250	8	8.05	135452250		7.49E-04	0.024	C	0.211	-0.264
S8_142226742	8	8.05	142226742	GRMZM2G050485_T01	3.70E-04	0.027	C	0.299	0.315
S8_160609980	8	8.06	160609980	GRMZM2G447406_T01	7.09E-04	0.024	A	0.076	-0.545
S8_164974058	8	8.06	164974058	GRMZM2G340319_T01	8.90E-04	0.023	C	0.496	0.344
S8_165158360	8	8.06	165158360	GRMZM2G316214_T01	4.48E-04	0.026	G	0.274	0.330
S8_165183086	8	8.06	165183086	GRMZM2G316362_T01, GRMZM2G316362_T02, GRMZM2G316362_T03	2.93E-04	0.028	T	0.382	-0.301

Marker	Chr.	bin	Position (bp)	Gene Model	P-Value	Proportion of Variance Explained	Minor Allele (Test Allele)	Minor Allele Frequency	Allele effect
S8_171150791	8	8.08	171150791	GRMZM5G806622_T01	5.13E-04	0.026	G	0.414	-0.409
S8_171275175	8	8.08	171275175		1.10E-04	0.032	A	0.214	-0.421
S8_172895746	8	8.08	172895746	GRMZM2G468132_T01	8.73E-04	0.024	A	0.239	0.346
S9_13150160	9	9.02	13150160	GRMZM2G116491_T01	4.58E-04	0.026	G	0.376	0.392
S9_37295487	9	9.03	37295487	GRMZM2G010637_T02, GRMZM2G010637_T01	8.80E-04	0.023	C	0.069	0.624
S9_118393797	9	9.04	118393797	GRMZM2G367985_T01	1.59E-04	0.030	A	0.067	0.432
S9_137288860	9	9.06	137288860	GRMZM2G700014_T01, GRMZM2G700014_T02	3.56E-04	0.027	G	0.309	-0.409
S9_151654313	9	9.07	151654313	GRMZM2G127072_T01	4.48E-04	0.026	G	0.410	0.273
S9_151819648	9	9.07	151819648	GRMZM2G010017_T02, GRMZM2G010017_T01, GRMZM2G010017_T03, GRMZM2G311232_T01	6.80E-04	0.024	T	0.075	0.688
S9_152760384	9	9.07	152760384	GRMZM5G820791_T01, GRMZM5G841893_T01, GRMZM5G841893_T02	4.01E-04	0.027	G	0.260	0.419
S9_155570200	9	9.08	155570200	GRMZM2G079066_T02, GRMZM2G079066_T01	1.64E-04	0.030	A	0.051	0.847
S9_155597023	9	9.08	155597023	GRMZM2G161905_T01	5.24E-04	0.026	A	0.053	0.497
S10_9213023	10	10.02	9213023	GRMZM2G544539_T01	9.70E-04	0.023	T	0.411	-0.237
S10_11602603	10	10.02	11602603	GRMZM2G413943_T01	6.86E-04	0.024	A	0.307	-0.130
S10_102133795	10	10.04	102133795		9.55E-04	0.023	G	0.059	0.586
S10_136883293	10	10.05	136883293		7.32E-04	0.024	C	0.307	0.325
S10_144682500	10	10.07	144682500	GRMZM2G136960_T01	2.29E-04	0.029	A	0.148	0.331
S10_144932244	10	10.07	144932244		7.26E-04	0.024	A	0.200	-0.282
S10_144934801	10	10.07	144934801		6.47E-04	0.025	T	0.476	0.301

Supplementary table 1b. Significant SNP associations for trait ME through MLM

Marker	Chr	bin	Position (bp)	Gene Model	P-Value	Proportion of Variance Explained	Minor Allele (Test Allele)	Minor Allele Frequency	Alleleic effect
S1_4724733	1	1.01	4724733	GRMZM2G105436_T01	8.46E-05	0.033	G	0.292	0.050
S1_173234207	1	1.05	173234207	GRMZM2G003509_T01, GRMZM2G003509_T02	1.87E-04	0.030	G	0.496	-0.043
S1_226456940	1	1.07	226456940	GRMZM2G131817_T01, GRMZM2G131817_T02	2.08E-04	0.029	C	0.248	0.048
S1_298273257	1	1.12	298273257	GRMZM2G159353_T01	2.57E-04	0.028	T	0.103	-0.088
S2_12243893	2	2.02	12243893		3.03E-04	0.028	C	0.450	-0.038
S2_12243894	2	2.02	12243894		1.80E-04	0.030	G	0.348	-0.044
S2_24450868	2	2.03	24450868	GRMZM2G064124_T01	3.23E-04	0.027	A	0.076	0.091
S2_30907772	2	2.04	30907772	GRMZM2G352129_T01	3.61E-04	0.027	C	0.251	-0.048
S2_30909942	2	2.04	30909942	GRMZM2G352129_T01	3.75E-04	0.027	T	0.229	-0.049
S2_39751613	2	2.04	39751613	GRMZM2G479018_T01	2.91E-04	0.028	C	0.082	-0.077
S2_44721856	2	2.04	44721856	GRMZM2G355906_T02, GRMZM2G355906_T01, GRMZM2G355906_T04, GRMZM2G355906_T03, GRMZM5G834459_T01	2.19E-04	0.029	A	0.054	-0.100
S2_83268098	2	2.05	83268098	GRMZM2G177575_T02, GRMZM2G177575_T03, GRMZM2G177575_T01, GRMZM2G177575_T04	2.96E-04	0.028	T	0.080	-0.109
S2_100778676	2	2.05	100778676		3.50E-04	0.027	A	0.070	0.106
S2_104735236	2	2.05	104735236	GRMZM2G149708_T02, GRMZM2G149708_T01, AC210605.3_FGT003	3.31E-04	0.027	C	0.167	-0.080
S2_131425963	2	2.05	131425963	AC211746.3_FGT006	8.70E-06	0.042	C	0.291	-0.054
S2_131425968	2	2.05	131425968	AC211746.3_FGT006	4.60E-05	0.035	G	0.295	-0.049
S2_201966646	2	2.07	201966646	GRMZM2G113794_T01, GRMZM5G858373_T01	2.99E-04	0.028	A	0.209	0.055
S2_235226296	2	2.09	235226296	GRMZM2G178136_T01	9.58E-05	0.032	C	0.307	-0.070
S2_235226332	2	2.09	235226332	GRMZM2G178136_T01	2.39E-04	0.029	C	0.298	-0.069

Supplementary table 1b. Significant SNP associations for trait ME through MLM

Marker	Chr	bin	Position (bp)	Gene Model	P-Value	Proportion of Variance Explained	Minor Allele (Test Allele)	Minor Allele Frequency	Alleleic effect
S3_149247409	3	3.05	149247409	GRMZM5G833593_T03, GRMZM5G833593_T04, GRMZM5G833593_T05, GRMZM5G833593_T01, GRMZM5G833593_T02	2.38E-04	0.029	T	0.158	0.055
S3_168012590	3	3.05	168012590	GRMZM2G392710_T01, GRMZM2G392710_T02	2.27E-04	0.029	C	0.396	-0.034
S3_168838080	3	3.06	168838080	GRMZM2G156861_T03, GRMZM2G156861_T01, GRMZM2G458554_T01	3.74E-04	0.027	A	0.062	0.085
S3_212406341	3	3.08	212406341	GRMZM5G822563_T03, GRMZM5G822563_T02, GRMZM5G822563_T01	1.48E-04	0.030	T	0.071	-0.076
S3_227586232	3	3.09	227586232	GRMZM2G035785_T01	2.28E-04	0.029	C	0.172	-0.083
S3_227586233	3	3.09	227586233	GRMZM2G035785_T01	2.97E-04	0.028	G	0.174	-0.084
S3_227586235	3	3.09	227586235	GRMZM2G035785_T01	9.13E-05	0.032	G	0.176	-0.084
S3_227586237	3	3.09	227586237	GRMZM2G035785_T01	2.28E-04	0.029	A	0.172	-0.083
S4_182947054	4	4.08	182947054	GRMZM2G011469_T01, GRMZM2G011469_T02	3.89E-04	0.027	T	0.069	-0.051
S4_238275507	4	4.1	238275507		1.71E-04	0.030	C	0.261	-0.075
S4_238275508	4	4.1	238275508		1.71E-04	0.030	A	0.261	-0.075
S4_238275759	4	4.1	238275759		3.53E-04	0.027	T	0.479	-0.052
S4_238275784	4	4.1	238275784		2.38E-04	0.029	G	0.492	-0.054
S4_238357174	4	4.1	238357174	GRMZM2G423137_T02, GRMZM2G423137_T01, GRMZM5G878640_T02, GRMZM5G878640_T01	2.30E-04	0.029	A	0.170	0.070
S5_2188003	5	5	2188003	GRMZM2G085336_T01, GRMZM2G085336_T02	2.69E-04	0.028	G	0.402	-0.057

Supplementary table 1b. Significant SNP associations for trait ME through MLM

Marker	Chr	bin	Position (bp)	Gene Model	P-Value	Proportion of Variance Explained	Minor Allele (Test Allele)	Minor Allele Frequency	Alleleic effect
S5_2400437	5	5	2400437	GRMZM2G002765_T04, GRMZM2G002765_T02, GRMZM2G002765_T03, GRMZM2G002765_T01, GRMZM5G860816_T01	6.31E-05	0.034	A	0.391	-0.054
S5_200435108	5	5.06	200435108	GRMZM2G325019_T01	2.14E-05	0.038	A	0.308	-0.053
S5_200437468	5	5.06	200437468	GRMZM2G325019_T01	1.54E-04	0.030	C	0.372	-0.050
S5_215984446	5	5.09	215984446	GRMZM2G364172_T03, GRMZM2G364172_T02	3.26E-04	0.027	C	0.317	-0.031
S6_2043652	6	6	2043652	GRMZM2G009627_T01	1.18E-04	0.031	C	0.311	-0.042
S6_44821882	6	6.01	44821882		2.42E-04	0.029	C	0.395	-0.049
S6_44821884	6	6.01	44821884		2.81E-04	0.028	G	0.396	-0.049
S6_44821885	6	6.01	44821885		2.81E-04	0.028	C	0.396	-0.049
S6_44821886	6	6.01	44821886		2.81E-04	0.028	C	0.396	-0.049
S6_44821933	6	6.01	44821933		2.81E-04	0.028	C	0.396	-0.049
S6_45183186	6	6.01	45183186	GRMZM2G124617_T02, GRMZM2G124617_T01	3.51E-04	0.027	G	0.380	-0.051
S6_73801877	6	6.01	73801877	AC209946.4_FGT002	3.80E-04	0.027	T	0.473	-0.045
S6_93495717	6	6.02	93495717	GRMZM2G161666_T01	1.29E-04	0.031	C	0.179	0.069
S6_147868122	6	6.05	147868122		2.04E-04	0.029	G	0.272	-0.062
S6_165945806	6	6.07	165945806	GRMZM2G520927_T01, GRMZM2G062738_T01	1.62E-04	0.030	C	0.440	-0.042
S7_69997119	7	7.02	69997119		1.56E-04	0.030	T	0.159	-0.061
S7_131438148	7	7.03	131438148	GRMZM2G018108_T01, GRMZM2G018108_T02	2.95E-05	0.037	A	0.130	0.090
S7_132169495	7	7.03	132169495		2.56E-04	0.028	G	0.104	0.102
S7_151404619	7	7.03	151404619		2.50E-04	0.028	C	0.215	-0.048
S7_165798736	7	7.04	165798736		9.39E-05	0.032	T	0.217	-0.061
S7_167348106	7	7.04	167348106	GRMZM2G158130_T01	1.13E-04	0.032	A	0.101	0.082
S8_68087278	8	8.03	68087278		1.52E-04	0.030	A	0.061	0.111
S8_101506781	8	8.03	101506781		3.30E-04	0.027	C	0.426	-0.033
S8_142226742	8	8.05	142226742	GRMZM2G050485_T01	2.51E-04	0.028	C	0.299	0.053

Supplementary table 1b. Significant SNP associations for trait ME through MLM

Marker	Chr	bin	Position (bp)	Gene Model	P-Value	Proportion of Variance Explained	Minor Allele (Test Allele)	Minor Allele Frequency	Alleleic effect
S8_165183086	8	8.06	165183086	GRMZM2G316362_T01, GRMZM2G316362_T02, GRMZM2G316362_T03	2.72E-04	0.028	T	0.382	-0.050
S8_171150791	8	8.08	171150791	GRMZM5G806622_T01	3.65E-04	0.027	G	0.414	-0.068
S8_171275175	8	8.08	171275175		1.53E-04	0.030	A	0.214	-0.069
S9_13150160	9	9.02	13150160	GRMZM2G116491_T01	2.63E-04	0.028	G	0.376	0.066
S9_118393797	9	9.04	118393797	GRMZM2G367985_T01	2.70E-04	0.028	A	0.067	0.073
S9_155570200	9	9.08	155570200	GRMZM2G079066_T02, GRMZM2G079066_T01	1.61E-04	0.030	A	0.051	0.143

SupplementaryTable 2. Predicted values of the two traits IVOMD and ME for the DH lines based on the prediction model set of 156,884 SNPs for the two traits

Parameter	IVOMD (%)	ME (MJ/Kg)
Mean of top 50	48.9	7.22
Range of top 50	48.79 to 49.27	7.20 to 7.27
Mean of bottom 50	47.63	7.02
Range of bottom 50	47.22 to 47.8	6.96 to 7.05
Mean of all DH lines	48.43	7.15
Range of the DH lines	47.22 to 49.27	6.96 to 7.27

Supplementary table 3. Prediction accuracy between high and low group as determined by Pearson's coefficient between predicted value estimated at different marker density (sampled 20 times) and the observed phenotype using the training set of advanced related breeding lines

Marker density (SNPs)	IVOMD (%)		ME (MJ/Kg)	
	Low	High	Low	High
200	0.00	0.15	0.07	0.17
500	0.06	0.19	0.00	0.17
1000	0.00	0.24	0.05	0.36
3000	0.02	0.29	0.08	0.26
5000	0.06	0.31	0.05	0.30
10000	0.08	0.39	0.05	0.36
50000	0.08	0.55	0.07	0.47
100000	0.08	0.57	0.05	0.51
156884	0.08	0.57	0.06	0.52

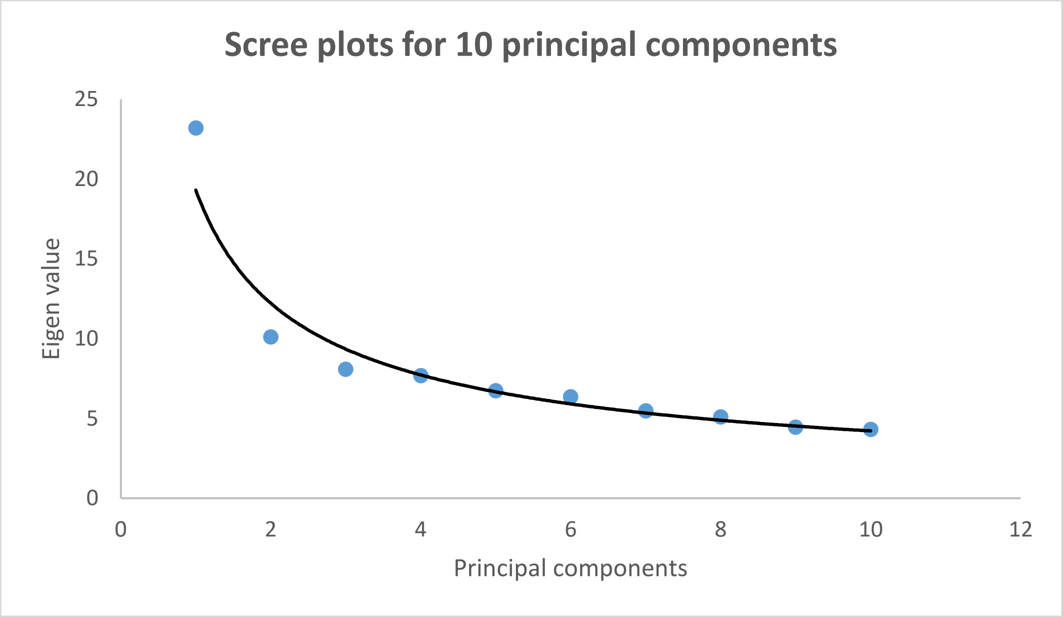

Supplementary figure 1. Scree plot representing 10 principal components
